# The incidence of psoriasis among smokers and/or former smokers inflammatory bowel diseases patients treated with tumor necrosis factor antagonist: A systematic review and meta-analysis

**DOI:** 10.1097/MD.0000000000027510

**Published:** 2021-10-22

**Authors:** Meiqi Yang, Weixin Liu, Qiuping Deng, Zeng Liang, Qin Wang

**Affiliations:** The First Affiliated Hospital of China Medical University, No. 155, Beier Road, Nanjing Street, Shenyang City, Liaoning Province, China.

**Keywords:** adalimumab, effectors, inflammatory bowel disease, infliximab, psoriasis, TNF-antagonist

## Abstract

**Background:**

Infliximab (IFX) and adalimumab (ADA) refer to the classic drugs to treat moderate-severe inflammatory bowel disease (IBD), which have been proven to be effective to control IBD. However, the side effects exerted by IFX and ADA should be monitored in therapies, especially the paradoxical reaction of the skin system (e.g., psoriasis). Psoriasis is recognized as the most common skin lesion, capable of significantly affecting the quality of patients’ life.

**Methods:**

This study searched literatures published in English language with the qualifications on PubMed, Embase, Web of Science, Google, and Geenmedical databases. Over 2 co-authors assessed the quality of the articles and extracted the data independently. The data acquired were statistically analyzed with the statistical software of Revman and Stata.

**Results:**

The ADA Group achieved a higher incidence of psoriasis (odds ratio [OR] = 0.658, 95% confidence interval [CI] [0.471–0.919]); Females achieved a higher incidence of psoriasis than males (OR = 1.941, 95%CI [1.326–2.843], *P* < .05); Smoking up-regulated the incidence of psoriasis (OR = 1.679, 95%CI [1.237–2.279], *P* < .05); The interval of medication was over 1 year, and the interval of medication applying IFX was longer than that of the ADA Group; most cases could be relieved by using local hormone, phototherapy, or systemic hormone therapy under the strategy of biological agents.

**Conclusions:**

The frequency of reported in IBD exceeds those of other autoimmune diseases, and the ADA treatment for IBD is safer than IFX. Psoriasis is more common in females than in males. Smoking refers to one of risk factors of psoriasis.

## 1 Introduction

Inflammatory bowel disease (IBD) refers to a chronic, non-specific inflammatory disease attributed to autoimmune disturbance of the intestinal mucosa, which can cause recurrent inflammatory lesions.^[1,2]^ The pathogenesis of IBD consists of the presence of dysfunctional gut microbiota, immune response dysregulation, environmental variations, and gene variants.^[3]^ On the whole, IBD comprises ulcerative colitis (UC), Crohn disease (CD), and undifferentiated types, severely affecting the quality of life of patients and generally requiring ongoing combination therapy. Tumor necrosis factor-C (TNF-α) antagonists are critical to treating a wide range of autoimmune inflammatory diseases (e.g., rheumatoid arthritis [RA], IBD, and psoriasis).^[4]^ As suggested from the 2018 Inflammatory Bowel Disease Consensus, biological agents should be considered to treat moderate to severe UC.^[5]^ Given the American Gastroenterological Association clinical guidelines, for patients with moderate to severe diseases and not responding to mesalazine, hormonal, or immunosuppressive agents, the use of biological agents should be considered.^[6]^ TNF-α is expressed in considerable intestinal mucosal cells in IBD patients, directly involved in the disease occurrence and progression.^[1]^ TNF-α-antagonists have been proven to be effective for refractory UC and CD patients with fistula and sinus formation.^[2]^ The efficacy of TNF-α-antagonists in respect of IBD treatment has been confirmed.^[7]^ As TNF-α-antagonists have been increasingly employed, the occurrence of drug-induced side effects cannot be ignored. Psoriasis refers to an autoimmune disease seriously affecting the quality of people's daily life, generally occurring after treatments with TNF-α-antagonists.^[8,9]^ Psoriasis is attributed to a complex mechanism between the immune system, psoriasis autoantigens, inflammatory cytokines, as well as multiple environmental factors.^[10]^ Besides, there have been cases of pathogenic infection, vasculitis, drug induced lupus, eczema, erythema multiform, and a wide range of skin malignancies.^[11]^ TNF-α is considered the vital factor in the inflammatory reaction by regulating the inflammatory signal transduction pathway (e.g., TNF pathway). TNF-α-antagonists have been extensively employed for treating psoriasis. However, with the increase in contradictory reactions, the safety of TNF-α-antagonists should be monitored. In addition, psoriasis is an autoimmune skin disease with abnormal T cell-mediated keratinocytes overly proliferated and abnormally differentiated.^[12–14]^ According to existing studies, TNF-α-antagonist-induced psoriasis and primary psoriasis are not identical in histopathology and immunohistology. A proportion of meta-analysis was employed to study the correlation between psoriasis and IBD, as an attempt to prove the significant bidirectional correlation between them.^[15]^

At present, the most extensively employed TNF-antagonists include infliximab (IFX) and adalimumab (ADA). IFX and ADA are TNF-α antagonists capable of inhibiting TNF-α production and exertion. IFX and ADA are broadly applied in autoimmune diseases (e.g., IBD, psoriasis, and RA). IFX is initially used for treating adult and juvenile IBD, and ADA has been primarily utilized in adult IBD.^[15]^ The prevalence of TNF-α antagonists induced psoriasis between different biological agents and different genders and smokers remains controversial. In accordance with the published literatures, this study found that the smokers and ex-smokers are more prone to psoriasis and IBD. This study aimed to describe the prevalence and correlation between psoriasis and usage of biological agents and relevant risk factors in IBD patients by conducting a systematic review and meta-analysis.

## 2 Methods

### 2.1 Search strategy

This study was conducted by complying with the Preferred Reporting Items for Systematic Reviews and Meta-Analyses (PRISMA). By collecting 2000-to-date literatures in PubMed, Embase, Web of Science, and other databases, the key words for searching were set, that is, Inflammatory Bowel disease, IBD, Chron's disease, CD, Ulcerative colitis, UC, psoriatic, PSO, paradoxical reaction, adverse reaction, IFX, ADA, as well as biological agents. For the search in title/abstract, by searching keywords (e.g., “and”, “or”) and by using Medical Subject Headings keywords and advanced search, some references with high quality were also checked, and the literatures meeting the standard were screened out. After the preliminary screening, over 500 literatures were initially taken. The literatures were further screened based on the inclusion and exclusion criteria.

### 2.2 Inclusion and exclusion criteria

Literatures satisfying the inclusion criteria below were included in the meta-analysis: It was observational; the data were complete, and the incidence of psoriasis was clear; for a case-control study, there was complete information from the experimental and controls.

Exclusion criteria of the literatures: subjects with other autoimmune diseases or suffering from more severe systemic diseases; the experiment poorly designed or lacking a control; psoriasis was not pathologically confirmed; the score of the article was lower than 6 after the assessment of the quality of Newcastle-Ottawa Scale (NOS); the identical article published repeatedly.

By summarizing the conclusions of the respective study, an analysis was conducted on the incidence, the distribution of psoriasis, the time interval between onset and medication, as well as the prognosis of psoriasis. The required data were extracted: general data, that is, first author's name, year of publication, population nationality, inclusion sample size, gender distribution, distribution of IFX and ADA use, as well as smoking history; outcome data, that is, sample size, adjuvant therapy, lesion coverage, and management of psoriasis in the psoriasis group and the control (Table 1). The NOS assessment scale was adopted to assess the quality of the collected data.

**Table 1:** Characteristics of included literatures.

					Gender					Smoke history		Type of anti-TNF					
					Psoriasis		Control										
Authors	Nation	Age	Time between initiation of anti-TNF and onset of the cutaneous reaction	Psoriasis	M	F	M	F	Rash distribution	PSO	All	IFX	All	ADA	All	Commitant therapy	Number of discontinued anti-TNF therapy
Kirthi et al	Dublin	2017	No mention	N = 8	5	3	699	652	No mention	3	1384	2	237	6	166	n = 4 with azathioprine	No mention
Sridhar et al	Culunmbus	2018	14.6 m (CD), 11.6 m (UC)	N = 33	14	19	202	160	No mention	No mention		28	303	5	101	Thiopurines (5), Methotrexate (5)	N = 3
Rahier et al	Europe	2010	17 m (IFX), 12 m (ADA)	N = 62	20	42	3	20	Scalp (46%), umbilicus (31%), extremitis (31%), face + posttauricular (21%)	28	38	39	60	15	20	None	N = 28
Guerra et al	Spain	2012	14 m	N = 21	6	15	No mention		No mention	No mention		14	808	7	412	Thiopurines (12), thiopurines and oral steroids (2), aminosalicylates and oral steroids (1), methotrexate (1)	N = 4
Cleyne et al	Leuven	2016	22.8 m	N = 264	101	163	392	361	No mention	87	276	189		70		No mention	N = 2
Fréling	France	2015	38.7 m (IFX), 25.7 m (ADA)	N = 59	No mention		227	356	No mention	38	51	529	8	54	No mention	N = 32
Hiremath et al	Inova	2011	21 m	N = 6	2	4	41	26	Facial (5), perineum (1); plaque (1), popular (3), scaly (2)	No mention		73	73			No mention	N = 1
Guerra et al	Spain	2016	10 m (IFX), 12 m (ADA)	N = 125	48	77	3830	3460	Palms (48), scalp (41), limbs (41), folds (26), trunk (22), genitals (14), facial and/or retro auricular (13)	69	31,311	77	5725	48	3455	Thiopurunes (52), methotrexate (10)	N = 38
Mälkönen et al	Finland	2014	12 m	N = 40	23	17	28	16	No mention	No mention		84	No mention			5-ASA (8), glucocorticoid (3), azathioprine or methotrexate (1)	N = 7
George et al	Caucasi	2015	58 w	N = 18	4	14	29	43	Palmo-plantar (53%), trunk (47%), scalp (53%)	12	45	13	37	5	20	Azathioprine (6), cocorticoid (2)	N = 9
Afzali et al	American	2013	31.6 m	N = 17	8	9	No mention		Palmoplantar = 8, flexural = 1, prediction sites = 18	No mention		8	620	10	243	Azathioprine (3), 6MP (1), oralmethotrexate (5), sqmethotrexate (1), NAP (1)	N = 11
Andrade et al	Porto	2016	53 m	N = 39	10	29	371	322	Palmoplantar = 12, scalp = 11, trunk = 10, folds = 8, generalized = 2	21	193	20	473	19	220	Immunosuppression (135), corticoid (10)	N = 2

### 2.3 Statistical analysis

The odds ratio (OR) value was determined with the random effect model method, and the incidence ratio between the experimental group and the control was estimated. In addition, the heterogeneity between the studies was obtained by performing Cochrane Q-test and I^2^ static,^[16]^ in which I^2^ denotes the total variation across studies due to heterogeneity rather than chance. The equation of I^2^ is expressed as:


$${\text{I}}^{2}=100\%*(\text{Q-df})/\text{Q}$$

Q denotes the Cochran heterogeneity data; df represents the degree of freedom. The funnel plot was employed to analyze the publication bias. However, as impacted by the small number of articles and strict quality assessment, combined with the funnel plots, it could be considered that no publication bias was identified. Revman and Stata, version 12.0, were applied for all data analysis. *P* < .05 was considered with statistical significance.

## 3 Result

### 3.1 Study characteristics

**Figure 1:**
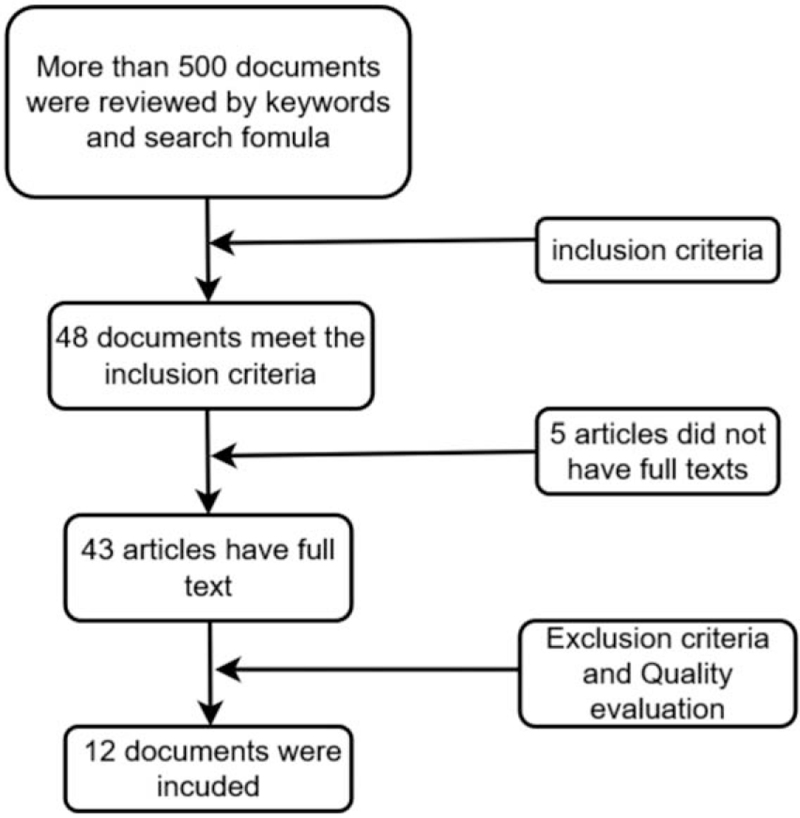
Flow diagram of the literatures screening.

Specific description of screening steps (Fig. 1): over 500 studies were obtained by preliminarily screening topics and abstracts. Of the mentioned results, 48 studies complied with the inclusion criteria, from which 5 studies that could not obtained in full text. After the full texts were read, 27 studies were excluded based on the exclusion criteria. After the quality assessment again, only 12 studies were finally selected in this meta-analysis. Table 1 lists the characteristics of the 12 literatures. The year of publication of all the studies ranged from 2010 to 2018. The data of the nation, age, interval, rate of psoriasis in IBD, gender, rash distribution, smoke history, type of TNF-antagonist, and medication condition were collected. After the data were analyzed, all the results were pooled together in Table 2.

**Table 2:** Summary of the results.

	Subgroup	Number of studies	Summary OR (95% CI) random effect model	Q	P-heterogeneity	I^2^ statistic%
Drug		8	0.658 (0.471–0.919)	11.55	0.172	30.7%
Gender		9	1.941 (1.326–2.843)	19.22	0.014	58.4%
	Pediatric	4	2.087 (1.329–3.277)	3.23	0.358	7.0%
	Adults	5	1.911 (1.085–3.366)	14.57	0.006	72.5%
Smoke		6	1.679 (1.237–2.279)	7.22	0.205	30.8%
	Small	2	1.528 (0.706–3.307)	1.14	0.285	12.7%
	Large	4	1.743 (1.1899–2.556)	6.06	0.109	50.5%

### 3.2 Summary of the results

#### 3.2.1 Comparison of the incidence of psoriasis induced by IFX and ADA

**Figure 2:**
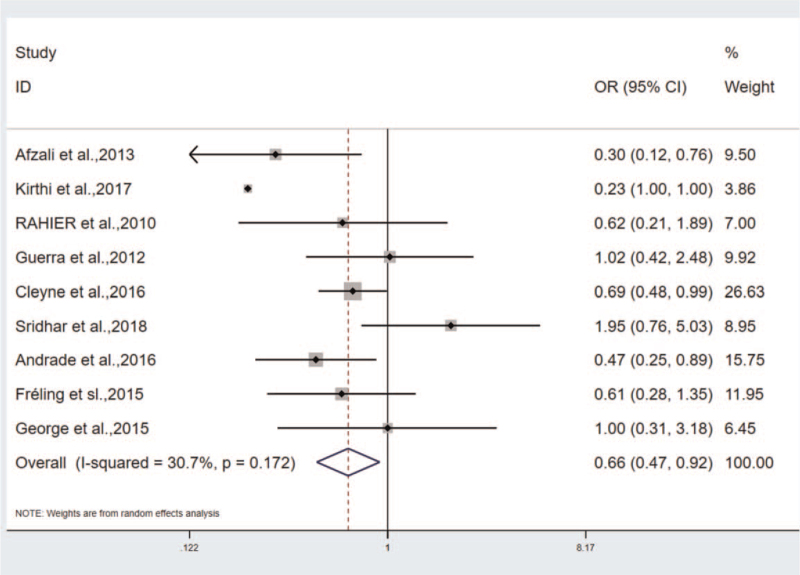
The correlation between infliximab and adalimumab groups.

The patients administrated with IFX and ADA were considered the experimental group and the observation group, respectively. The incidence of psoriasis was compared between the 2 drugs. According to the results, OR = 0.658, 95%CI (0.252–0.905), *P* < .05. The incidence rate of the IFX group was 0.658 times than the ADA group, heterogeneity: Q = 11.55, *P* = .172, I^2^ = 30.7% (Fig. 2). According to the further sensitivity analysis to identify heterogeneous sources, Afzali et al's study may contribute to the significant heterogeneity of the results. The heterogeneity rapidly decreased after the removal of the article: OR = 0.709, 95%CI (0.516–0.973), heterogeneity results: Q = 8.76, *P* = .271, I^2^ = 20.1%. Accordingly, Afzali et al's study can be considered the source of heterogeneity.^[17]^ No significant difference in the incidence and heterogeneity of psoriasis was identified in the sensitivity analysis of other studies.

#### 3.2.2 Effect of sex on the incidence of psoriasis induced by TNF-α-antagonist

**Figure 3:**
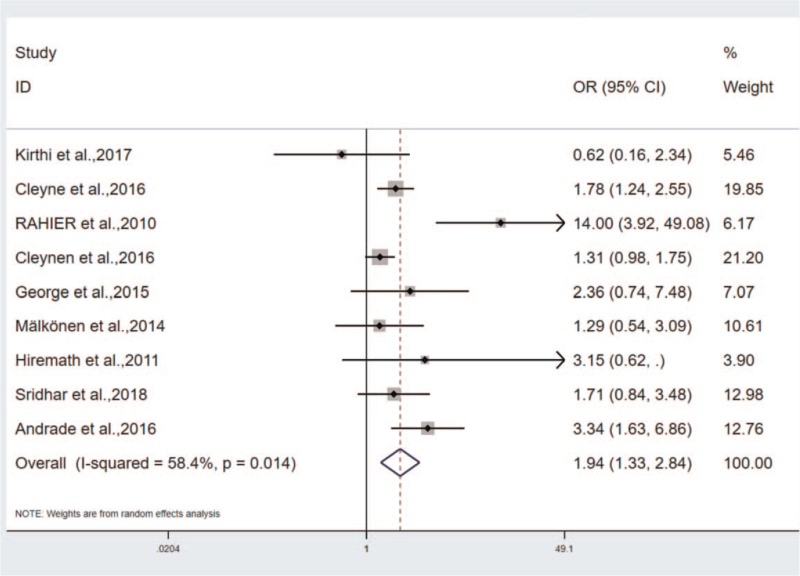
The correlation between different gender groups.

The incidence of psoriasis in different gender groups of IBD including 9 studies, respectively. The incidence of different gender groups after the treatment with TNF-antagonist, and the results were statistically significant: OR = 1.941, 95%CI (1.326–2.843), *P* < .01. The heterogeneity results: Q = 19.22, *P* = .014, I^2^ = 58.4% (Fig. 3). As indicated from the further sensitivity analysis, OR value of Rahier et al., the study was lower (OR = 14, 95%CI [3.917–49.081]), probably affecting the stability and reliability of the results and increasing the heterogeneity of the results. After excluding the study, the results were analyzed again. As revealed from the results, the heterogeneity decreased significantly (Q = 8.96, *P* = .256, I^2^ = 21.9%). The subjects in this study were primarily patients with CD, and the observation groups were patients with other skin diseases besides psoriasis. Some patients might have multiple types of skin lesions simultaneously, so there may be crossed interaction between various skin lesions.

**Figure 4:**
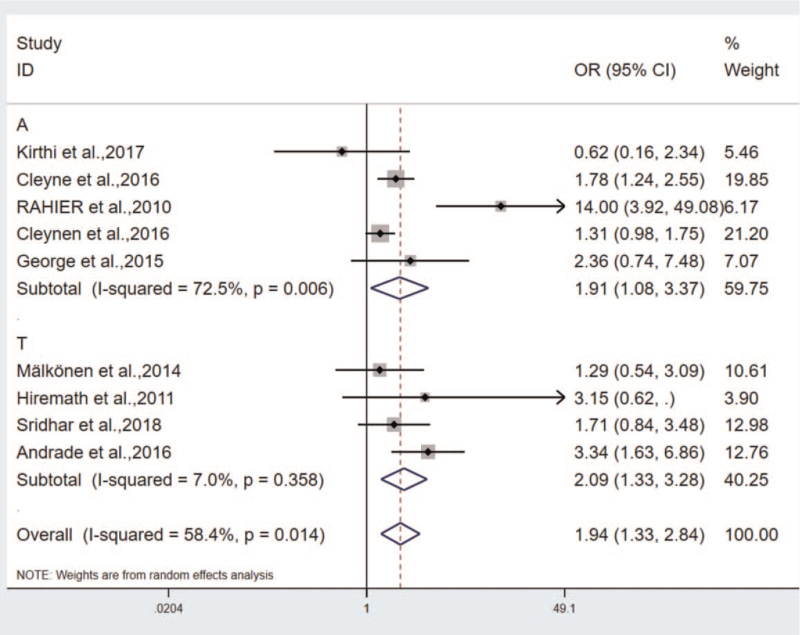
The subgroups analysis between different gender groups: Adults group and Teenagers group.

The study fell to 2 subgroups, that is., the adult group and the teenager group. The results included: OR_Adult group_ = 1.911, 95%CI (1.085–3.366), *P* < .05, heterogeneity: I^2^ = 72.5%, OR_Teenager group_ = 2.087, 95%CI (1.329–3.277), *P* < .05, heterogeneity: I^2^ = 7.0% (Fig. 4). According to the 2 subgroups, the conclusion was the same: Psoriasis is higher in females, and the OR_Teenager group_ > OR_Adult group_. The conclusion was drawn that the incidence of psoriasis is higher in the pediatric IBD patients administrated with TNF-antagonist.^[22]^

#### 3.2.3 The effects of smoking on psoriasis

**Figure 5:**
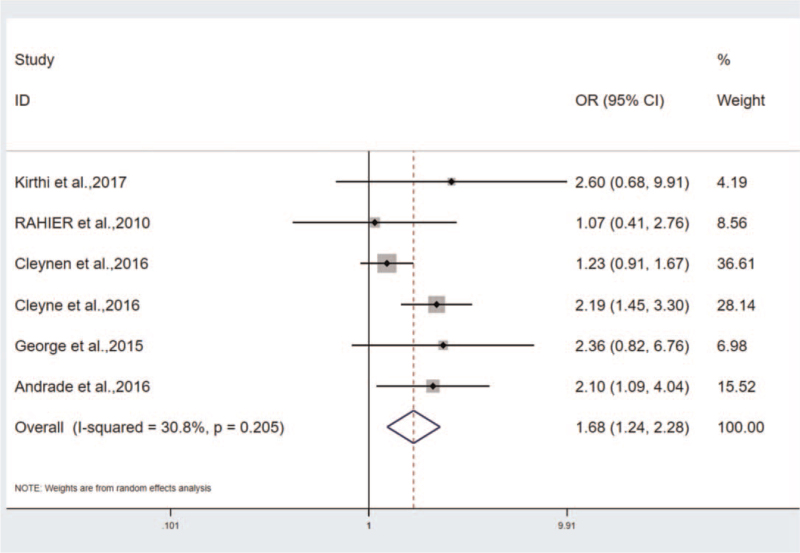
The correlation of incidence of psoriasis between smokers and non-smokers.

As revealed from the results, the incidence of psoriasis in the smoking/ex-smoking group were significantly higher than that in the non-smoking group, that is, OR = 1.679, *P* < .01, 95%CI (1.237–2.279), I^2^ = 30.8%) (Fig. 5). Psoriasis is more common in smokers/ex-smokers of IBD that were treated with TNF-antagonists. Smoking was reported as a risk factor for TNF-antagonists-induced psoriasis,^[26]^ which up-regulated the incidence of psoriasis, whereas some researchers suggested no significant difference between smokers and non-smokers in the incidence of psoriasis induced by TNF-antagonists.^[27]^ Accordingly, a conclusion might be drawn that smoking acts as a major risk factor for IBD, especially for UC patients. Quitting smoking refers to one of the interventions for IBD patients to control disease progression and prevent disease recurrence.

#### 3.2.4 Distribution of lesions

Statistically, skin lesions usually do not occur as a single form, and multiple skin lesions can occur simultaneously.^[28]^ Over 80% of the reported TNF-α-antagonist-induced psoriasis lesions involved more than 2 sites of body.^[14,23,24]^ As indicated from the existing literature, the skin lesions of psoriasis induced by TNF-α-antagonists were largely distributed in scalp, metacarpal, limbs, and skin folds.^[14,18,29,30]^ The most common lesions were located in the scalp and palmoplantar regions^[28]^ with the equal incidence, and the skin fold was found as the second favorable region.^[9]^ As reported from studies by Pugliese et al,^[31]^ the sacroiliac region, the extensor sides of the knee and elbow were the most frequently affected areas as well.

#### 3.2.5 Treatment and prognosis of psoriasis

It is currently considered that the treatment of psoriasis induced by TNF-α-antagonist should be assessed from the severity of psoriasis, the extent and degree of lesions, the impact on patients’ quality of life, and the effect on patients’ psychology, etc. Whether to use TNF-α-antagonist refers to a vital problem for treating psoriasis. The condition of patients and the variations of skin lesions should be monitored.^[32]^ It is not necessary to stop using TNF-α-antagonists in patients with mild psoriasis who have not yet significantly impacted normal quality of life.^[33,34]^ On the whole, local application of steroids is the preferred treatment for most lesions.^[17,35]^

#### 3.2.6 Pathogenesis of psoriasis

The occurrence of psoriasis is closely linked to autoimmune factors and may be correlated with genetic factors. All studies excluded subjects with personal and family history to ensure the reliability of the results. As suggested from some studies, psoriasis may be a prevalent side effect of TNF-α-antagonists, that is, class effects.^[11]^ The pathogenesis of psoriasis induced by TNF-α-antagonists remains unclear. According to existing reports, several mechanisms have been proposed. Under normal conditions, the balance of TNF-α and interferons (IFN)-γ is maintained in the body, contributing to the regulation of the body's inflammatory response. When TNF-α is significantly inhibited, the balance between TNF-α and IFN-γ is destroyed, thereby causing an increase in the production of IFN-γ by dermal plasmacytoma dendritic cell and excessively facilitating the migration of cluster of differentiation 8 + t cells to the epidermis; as a result, dendritic cell cells are activated and maturated, thereby inducing the autoimmune response.^[30,36]^ The interleukin (IL)-23/T-helper (Th)-17 axis is also vital to the pathogenesis of TNF-α-antagonist-induced psoriasis. IFN induced Th17 cell proliferation and IL-23 induced the release of large amounts of IL-17 from helper Th17 and Th1 cells, thereby disrupting cytokine balance in vita^[11]^ and then causing psoriasis,^[23,29,37]^ which may be correlated with IFN induced gene overexpression.^[21]^ Besides, there have been a small number of people considering that IBD patients with cytokine receptor mutation, thereby leading to psoriasis.^[17]^ The treatment with TNF-α-antagonists rises the risk of infection, and exposure to infected organisms elevates the risk of psoriasis, especially pustular psoriasis.^[33]^ Numerous autoimmune diseases originate at the gene level, TNF-α-antagonist-induced psoriasis may be correlated with the pathogenesis of psoriasis, and patients using TNF-α-antagonists are more genetic predisposition.^[38]^ Furthermore, it may be correlated with the genetic variation of IFN, and the role of the X chromosome in autoimmune diseases has been evidenced.^[39]^

### 3.3 Assessment of heterogeneity

**Figure 6:**
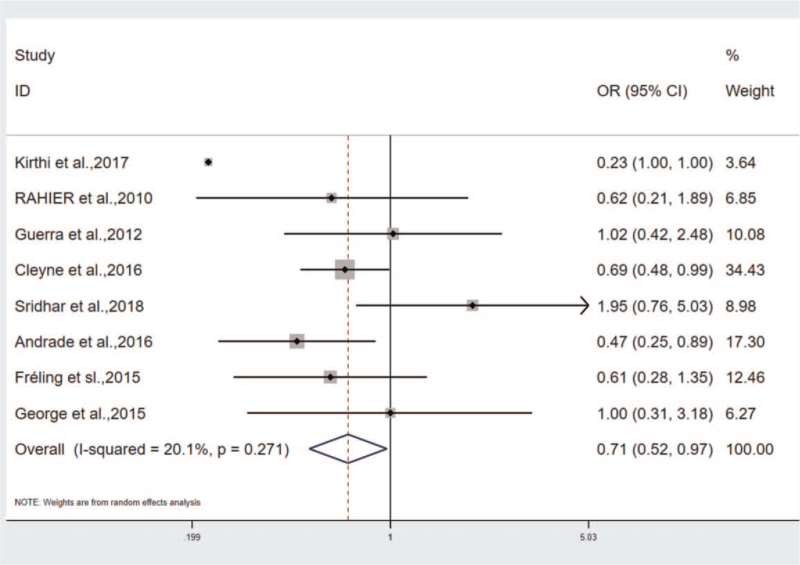
The heterogeneity analysis in the correlation between infliximab and adalimumab groups.

The heterogeneity was analyzed by conducting sensitivity analysis and subgroup analysis. In the analysis of correlation between IFX and ADA groups, Afzali et al primarily explored the drug-induced psoriasis in teenagers that led to the heterogeneity. After the study exclusion, the heterogeneity turned out to be Q = 8.76, *P* = .271, I^2^ = 20.1% (Fig. 6). Notably, this study is the source of heterogeneity. In the study on the correlation between gender and the incidence of TNF-antagonist induced psoriasis, subgroups were implemented by complying with age, and the 9 studies were divided into 2o groups, the adult group: OR = 1.911, 95%CI (1.085–3.366), *P* < .05, heterogeneity: I^2^ = 72.5%, the teenager group: OR = 2.087, 95%CI (1.329–3.277), *P* < .05, heterogeneity: I^2^ = 7.0% (Fig. 4). In the 2 groups, the incidence was higher in females, especially in the pediatric group. Thus, the heterogeneity primarily originates from the adult group.

**Figure 7:**
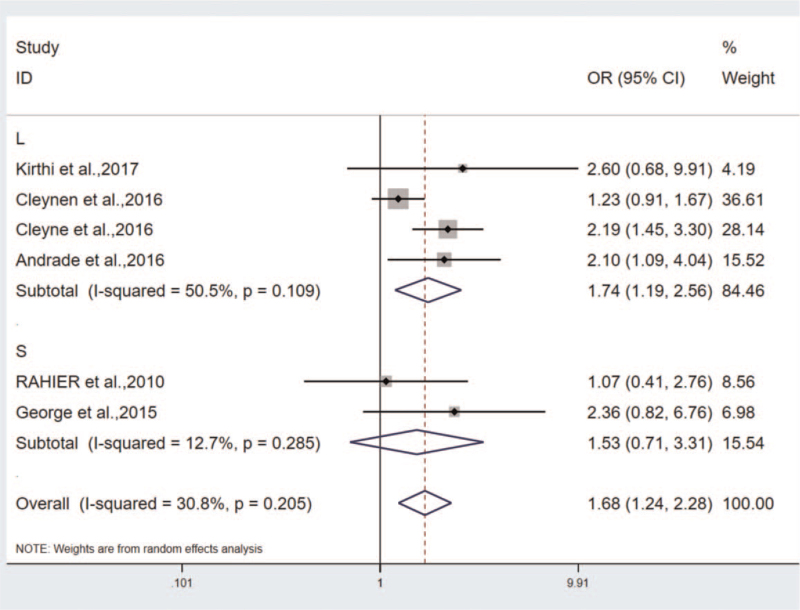
The subgroups analysis in the correlation of incidence of psoriasis between smokers and non-smokers.

In the study on smoking and drug-induced morbidity, the subgroup analysis was conducted by complying with the sample size, and the heterogeneity was found to mainly originate from the large sample size group: Q = 6.06, *P* = .109, I^2^ = 50.5%. Small sample size group: Q = 1.14, *P* = .285, I^2^ = 12.7% (Fig. 7). Since the heterogeneity may originate from the study of Guerra et al. and MD et al, both studies were removed, and the results were OR = 1.4, 95%CI (1.085–1.806), heterogeneity: Q = 4.00, *P* = .406, I^2^ = 0.0%.

### 3.4 Publication bias

**Figure 8:**
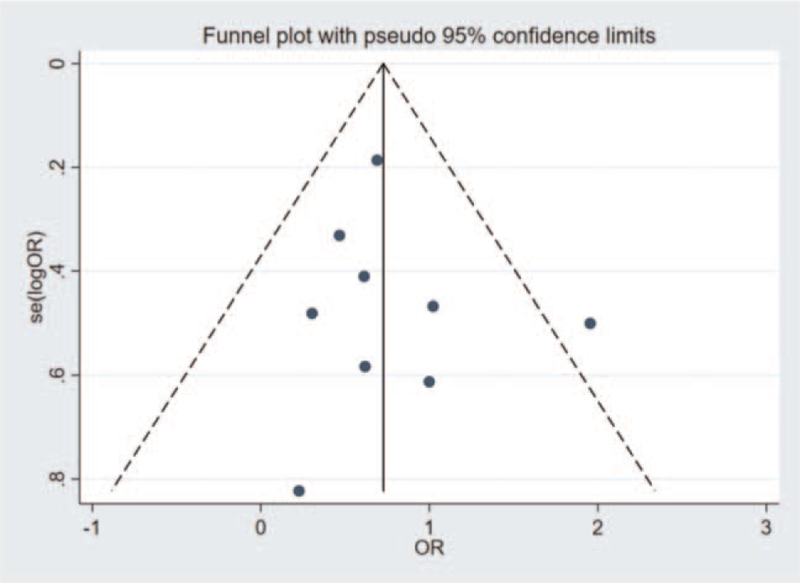
The funnel plot.

By conducting the funnel plots (Fig. 8), symmetrical distribution of the studies can be basically seen. The publication bias can be ignored.

## 4 Discussion

The present meta-analysis mainly analyzed the risk factors of psoriasis in IBD patients using the biological agents. IFX is the first biological agent applied in IBD, alone or in combination with immunomodulators or mesalazine. The basic characters of the studies are listed in Table 1. In the mentioned 12 articles, 10 of them originated from European nations, complying with the high incidence of IBD and the large usage of biological agents in European nations. The included studies were all published after 2010 year. First, by comparing the drug safety of IFX and ADA, this study found that the IFX group had a lower incidence of new onset psoriasis than the ADA group. It was therefore suggested that IFX is safer than ADA. The incidence of psoriasis in IFX was 0.66 times that of ADA, which might be correlated with the drug usage duration paradoxical reaction for patients having used IFX and ADA. The usage duration of IFX was longer than the ADA to treat CD than UC, especially in developing nations.^[13]^ According to existing studies, the median timespan between initiation of therapy to occurrence of psoriasis was 2 to 6 months.^[35]^ Genetic overlap between psoriasis and IBD may account for the distinction between IFX and ADA.^[40]^ The ability of selective function to inflammatory cytokines of biological agents and disruption of the cytokine milieu might be correlated with the occurrence of autoimmune diseases. However, as long as the ADA is more extensively employed in IBD patients, more attention will be paid on its safety, and novel studies reported the prevalence of adverse events to be higher in ADA than IFX.^[17,25]^

Second, the analysis concluded that females were predicted to be more prone to psoriasis than men in IBD (OR = 1.975) and had a 1.975 times higher incidence of psoriasis than males. Females are more predicted to be prone to autoimmune diseases (e.g., IBD, RA, and Graves disease). Moreover, women with IBD are more likely to develop psoriasis induced by TNF antagonist in considerable studies.^[19,29,31]^ In this study, to eliminate the effect of age, the mentioned studies were divided into 2 subgroups, that is, adult and teenager groups. In accordance with the results, the females of all groups were more prone to psoriasis than male, which proves that gender is a risk factor for psoriasis in IBD patients having used biological agents. The incidence of psoriasis was higher in females in all studies for pediatric patients, while only 1 study reported lower incidence in females.^[27]^ There were significant differences in the incidence of psoriasis in the different genders of the pediatric group (7–17 years) administrated with IFX.^[41]^ Besides, in most cohort studies on pediatric patients, TNF-d antagonist induced psoriasis was more predisposed to the female population.^[17,42]^ Third, IBD patients treated with TNF-α-antagonist had a higher incidence of psoriasis in smokers than that of non-smokers.^[11,14]^ Smoking has been identified as a major risk factor for TNF-antagonist-induced psoriasis among IBD patients,^[31]^ and smokers/ex-smokers are at high risk for drug-induced psoriasis. Lauren et al,^[23]^ discovered obesity as another risk factor for psoriasis besides smoking through the designed case-control study. The data results of the mentioned 3 parts were pooled into Table 2. The mechanism leading to the induction or exacerbation of psoriasis by TNF antagonists remains unclear.^[43]^ The change of therapy regimen is vital to prognosis in the presence of the TNF-α antagonists induced psoriasis. Ko et al^[44]^ concluded that the optimal choice for TNF-α antagonists induced psoriasis is discontinuation of biologic agents. Most IBD patients suffered from relapse or aggravation after the withdraw or switch of biological agents.^[45]^ However, some scholars considered that the continuation of biological agents would not affect the recovery of drug-induced psoriasis, and the lesions could recover under the treatment of the local hormone or topical dermatologic medicine. No adequate evidence has been found to prove that discontinuation of TNF antagonists for non-infectious skin lesions can alleviate condition.^[46]^ Pugliese et al^[31]^ initially reported that the combination of biological agents with immunosuppressors could down-regulate the risk of psoriasis.^[47]^

Of the 12 studies, a total of 692 patients suffered psoriasis induced by TNF-antagonists, and 137 patients stopped or switched to biological agents, and over 50% of them had good prognosis. Based on a 14-year single center large sample study, Freling et al^[20]^ concluded that about 50% of patients could control their conditions without stopping their medication through topical medicine, and approximately 20% should stop their medication. According to the statistics, nearly 40% of patients could relieve skin lesions through local hormones, while 20% of patients could relieve the condition through ultraviolet light therapy. Wolf et al investigated the clinical outcomes of patients in group 377 (754 cases) withdrawing or switching of TNF-α-antagonist and those continuing TNF-α-antagonist. The clinical recurrence rate was higher in 377 groups (754 cases) stopping or converting TNF-antagonist for non-medical reasons, and the frequency of in-hospital treatment, emergency treatment, and out-patient treatment was higher than that of the continued use of TNF-antagonist.^[37]^

It is noteworthy that psoriasis can also occur in IBD extraintestinal complications, which should be differentiated with TNF-α antagonists induced psoriasis.^[48]^ The pathogenesis has similarity between psoriasis and IBD. Drug-induced complications are reported to be more common than extraintestinal complications. In clinical practice, health education for patients with relapsed condition should be strengthened, and patients’ compliance should be improved. The long-term follow-up is required for IBD patients to ask for the effects and adverse events to make an accurate judgment and guide the subsequent regimens. Long-term management is advised to minimize the potential adverse effects of TNF antagonist therapy. The data sources all originated from the electronic medical records of different hospitals or databases which could be incomplete.

### 4.1 Conclusion

The incidence of psoriasis induced by TNF-α antagonists is higher than other autoimmune diseases, and IFX treatment for IBD is safer than ADA. The incidence of psoriasis is higher in females than in males, and the incidence of psoriasis is significantly higher in smokers/ex-smokers than that in non-smokers. Differences exist in various TNF-α antagonists, and the therapeutic strategy of TNF antagonists is not required to be changed in the event of psoriasis.

### 4.2 Limitation

All the literatures included in this meta-analysis were retrospective literatures. Although the 3 co-authors assessed the quality of the article separately through the NOS assessment list, the subjectivity of authors affected the quality assessment results. This study attempted to select the articles with complete data and screen out the low-quality literatures. Furthermore, all the studies included originate from European and American nations, and basically the studies of Asian and African nations were not contained.

## Author contributions

All authors have contributed to and approve the final version of the article. YMQ and LWX participated in the study design and wrote the main manuscript text, and DQP, LZ, and WQ participated in the data analysis. All authors reviewed the manuscript.

**Conceptualization:** Meiqi Yang, Weixin Liu.

**Data curation:** Meiqi Yang, Weixin Liu, Qin Wang.

**Formal analysis:** Meiqi Yang, Weixin Liu, Qin Wang.

**Investigation:** Meiqi Yang, Weixin Liu.

**Methodology:** Meiqi Yang, Weixin Liu.

**Writing – original draft:** Meiqi Yang, Qiuping Deng, Zeng Liang.

**Writing – review & editing:** Meiqi Yang.
